# Supplementary material for: Learning curve and surgical outcome of robotic assisted colorectal surgery with ERAS program
Source: Sci Rep. 2022 Nov 29;12:20566. doi: 10.1038/s41598-022-24665-w (PMC9709162; doi:10.1038/s41598-022-24665-w)
Supplement: Supplementary file 1 — Supplementary Information 1. [file 41598_2022_24665_MOESM1_ESM.zip › LSC Raw data-Ñ[▒K/054/OP2_ñw╝╨░O▒Kñσ.pdf]

正式摘要(電子簽章：000000179246997)

W83 / W83

臺中榮民總醫院

Operation Note

[redacted]

- 手術日期：2019/11/05
- 手術醫師：吳峰旭，Fellow 許家榮，CR 黃邑霖
- Anesthesia : GE
- Position : Supine
- Aseptic agents : Soap , Betadine Alcohol

Diagnosis

- Pre-operative diagnosis :gallstone
- Post-operative diagnosis : Ditto.

Surgical Indication

- for prevention of further biliary complication

Operation

- Laparoscopic Cholecystectomy

Finding

- gallbladder, in size of 5x2x2cm, without wall thickness. several tiny gallstones noted. Mild bile leakage was noted suspected due to iatrogenic injury.

Procedure

- GS was consulted for cholecytectomy. Subcyphoid port was added. Calot's triangel was dissected but mild hard to dissected. The gallbladder was deteched from the liver bed antegradely. Cystic artery was ligated and then cystic duct was also dissected. The gallbladder was put into the endo-bag. CRS man take over the futher anastomosis.
- Estimated blood loss: minimum
- 器械數量: 正確
- 紗布數量: 正確

編輯主治 2215H吳峰旭

正式摘要(電子簽章：000000179246997)

W83 / W83

doctorRank:B
